# Prevalence of Smoking among Medical Students in a Tertiary Care Teaching Hospital

**DOI:** 10.31729/jnma.5006

**Published:** 2020-06

**Authors:** Neharika Shrestha, Nikhil Shrestha, Suzit Bhusal, Asmita Neupane, Rakshya Pandey, Nita Lohala, Arpan Pratik Bhandari, Mandeep Kumar Yadav, Abhinav Vaidya

**Affiliations:** 1 Oxford University Clinical Research Unit-Nepal, Patan Academy of Health Sciences, Lalitpur, Nepal; 2 Kathmandu Medical College and Teaching Hospital, Kathmandu, Nepal; 3 Kathmandu University School of Medical Sciences, Dhulikhel, Nepal; 4 B&B Hospital, Lalitpur, Nepal; 5 Jyoti Hospital, Kathmandu, Nepal; 6 Department of Community Medicine, Kathmandu Medical College and Teaching Hospital, Kathmandu, Nepal.

**Keywords:** *medical student*, *prevalence*, *smoking*, *tobacco*

## Abstract

**Introduction:**

Tobacco smoking is one of the most important preventable risk factors for non-communicable diseases. It has been seen that medical students have a higher frequency of smoking compared to the general population. This study aims to determine the prevalence of smoking among third-year medical students in a tertiary care teaching hospital in Nepal.

**Methods:**

This descriptive cross-sectional study was conducted among the hospital’s third-year undergraduate medical students over a four-month period (October 2019 to January 2020). Ethical clearance was received from the Institutional Review Committee of Kathmandu Medical College and Teaching Hospital. The whole sampling technique was used to collect data. The Global Health Professional Students Survey questionnaire was used to collect data. Data analysis was done in the statistical package for social sciences.

**Results:**

The prevalence of current smoking among selected medical students of Kathmandu Medical College and Teaching Hospital is 34 (30.1%), majority male 26 (23%). Fifty-six (49.4%) of them had ever smoked cigarettes in their life, and 27 (23.9%) had their first cigarette in late adolescence. The number of students who used other forms of tobacco was comparatively lower i.e. 6 (5.3%). Many of the students 53 (46.9%) were exposed to second-hand smoke both at home and in public, while 18 (15.9) exposed only at public places, and 6 (5.3%) only at home.

**Conclusions:**

Our study has concluded that there is a notable prevalence of smoking among the participants. This points to the need for specific training sessions in their clinical years about smoking cessation for themselves and regarding counseling for patients.

## INTRODUCTION

Tobacco is attributed to be a significant cause of preventable chronic diseases, and a major risk factor for premature death.^1^ WHO states that 7 million deaths occur annually due to tobacco use, which is expected to rise to 8 million by the end of 2030.^2^ Currently, 80% of the 1 billion smokers worldwide are from developing countries.^3,4,5^ Health professionals play a crucial role in the prevention and cessation of tobacco use via techniques such as counseling.^6,7^

Providing cessation training to health professional students is a vital towards tobacco control, which will aid WHO’s Tobacco Control programs.^8^

But most health professional students in Afro-Asian developing countries have little-to-no training on tobacco cessation techniques.^9^Providing these trainings can have a positive impact on the future professional practice strengthening tobacco control. Although health professionals who themselves smoke, cannot be expected to persuade a patient to quit smoking.^10,11^

This study aims to estimate the prevalence of smoking and tobacco products used by third-year medical students and their attitudes and behaviour towards it and smoking cessation trainings.

## METHODS

This descriptive cross-sectional study was conducted among all third-year undergraduates of Kathmandu Medical College and Teaching Hospital after taking the ethical clearance from the Institutional ethical review Committee, over four months in October 2019 to January 2020.

All the third-year medical students enrolled currently in the medicine in the tertiary teaching hospital who gave consent were included in the study. Chronically absent students, those students who were seriously ill at the time of the study, also students other than those in the third year were not included in the study. Students were informed about the study and those willing to participate were given consent paper for signature. A self-administered validated Global Health Professional Students Survey (GHPSS) questionnaire, was used in this study, developed by the World Health Organization (WHO), the United States Centers for Disease Control and Prevention (CDC) and the Canadian Public Health Association.^12,13^

Current smokers were defined as someone who smokes any tobacco product, either daily occasionally, during the past 30 days according to the WHO definition of a “smoker”. Ever-smoker was defined as one who had ever once smoked in his lifetime. Other tobacco products were defined as chewing tobacco, snuff, bidis, hookah, cigar, or pipes. Non-smokers were defined as those who never smoked a cigarette in their lifetime.^14^

The WHO’s GHPSS Questionnaire is a validated tool for screening smoking among university students as stated by the paper published by Gualano MR, et al.^12^ The questionnaire was administered during regular class sessions in an anonymous, voluntary manner, according to the protocol. The questionnaire was distributed to the participants by a single investigator and the questionnaire was collected on the same day. Participants were informed about the anonymity of their personal information, as well as of their right to opt-out of the study, any time within the study period. Despite the whole sampling, the following bias could occur such as information bias, reporting bias, social desirability bias, and non-response bias. Such bias present in the study were minimized as possible.

Data collected was thoroughly checked for its completeness. Only those forms, which have complete data were included in the study. The data was then coded and double-entry done in the statistical package for the social sciences (SPSS) version 23.0. The data was processed and analyzed by using simple descriptive statistics; in terms of percentage and frequency.

## RESULTS

**Figure 1:**
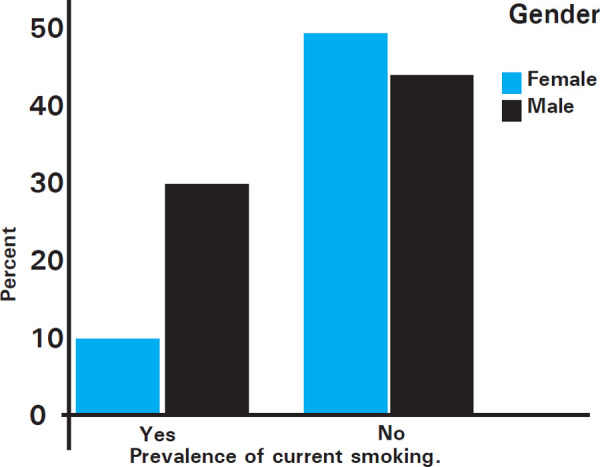
Prevalence of current smoking among third-year medical students.

The period prevalence of current smokers among third-year medical students of Kathmandu Medical College Teaching Hospital is 34 (30.1%). In the study we achieved a very good total response rate of 98%. Among the 113 participants, 56 (49.4%) were ever smokers and a major proportion of the students, 27 (23.9%) were first introduced to tobacco smoking in their late adolescent years (i.e. 20-24 years of age). The number of students using other forms of tobacco products such as chewing tobacco, snuff, bidis, hookah, cigar or pipes was proportionately lower that those smoking tobacco, i.e. 6 (5.3%) and 14 (12.4%) of the students had ever used the other forms of tobacco products (Figure 1).

In this study we found that the majority of current smokers 26 (23%w) were male, while only a small percent of the smokers were females 8 (7.1%). And most of the current smokers 25 (22.1%) were of the age group of 19-24 years, with few smokers 8 (7.1%) also of age group of 25-29 years.

**Figure 2:**
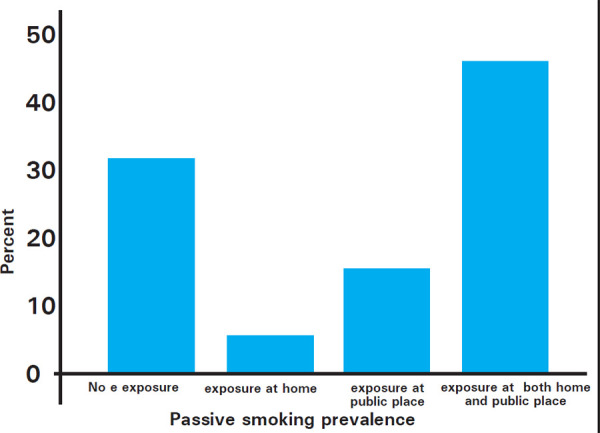
Prevalence of exposure to environmental tobacco smoke.

Non-smokers were more accepting of placing a ban on tobacco compared to smokers; whether it be banning sale to adolescents 69 (87.3%) versus (vs.) 26 (76.5%), banning tobacco advertisement 61 (77.2%) vs. 20 (58.8%), banning smoking in restaurants 62 (78.5%) vs. 15 (44.1%), banning smoking in disco/bars/pubs 43 (54.4%) vs. 10 (29.4%) or banning smoking in enclosed public places 70 (88.6%) vs. 25 (73.5% ). Among the participants the prevalence of exposure to environmental tobacco smoke at both home and public places was 53 (46.9%), exposure at home only was 6 (5.3%) and exposure at only public places was 18 (15.9%) (Figure 2).

Compared to smokers, non-smokers had a view that health professionals needed to get specific training on cessation techniques i.e. 76 (96.2%) in non-smokers vs. 27 (79.4%) in smokers and that health professionals who smoke are less likely to advise patients to stop smoking i.e. 52 (65.8%) in non-smokers vs. 14 (41.2%) in smokers.

No major differences in opinion regarding view on health professionals serving as “role models” for their patients and the general population 67 (84.8%) in non-smokers vs. 28 (82.4%) in smokers as well as, regarding the view that health professionals should routinely give advice on quitting smoking 76 (96.2%) in non-smokers vs. 34 (100.0%) in smokers. And both groups believed that health professionals had a role in giving advice about smoking cessation to patients 76 (96.2%) in non-smokers vs. 33 (97.1%) in smokers.

**Figure 3:**
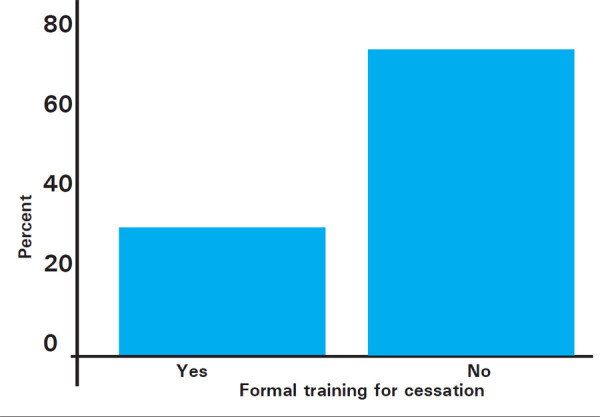
Participants who have received formal training for cessation.

Most of the students, 105 (92.9%) mentioned that the dangers of smoking were taught during the class. Similarly, 77 (68.1%) of students were also taught about the reason for people smoking. Also the majority of students 106 (93.8%) had learned about the importance of tobacco use history. About 87 (77%) of students had learned the importance of providing educational materials regarding quitting smoking to support cessation of tobacco use for those who want to quit smoking. Only 32 (28.3%) of the students actually received formal training regarding smoking cessation (Figure 3).

Most 100 (88.5%) of the participants knew about nicotine replacement therapies, and 68 (60.2%) were aware of the use of antidepressants for aiding cessation.

## DISCUSSION

Tobacco use is currently the major risk factor for non-communicable chronic illnesses, which is preventable.^15^ Health professionals play a pivotal role in the control of this existing problem. But, there are some crucial shortcomings; major being the health professionals themselves are prone to use tobacco.

In our study, the prevalence of smoking among third-year medical students was 30.1% which is lower than that of the general population (37%) and higher than that of adolescents (13.1%) at the national level.^16^ Using tobacco can not only risk the health of the medical students but will also undermine the credibility of the future health professional and his ability to deliver effective anti-tobacco counseling while managing future patients.^17^ Furthermore, cessation training if any provided to the health professionals proves to be inadequate, primarily in developing nations.^18,19^ This will most undoubtedly result in the inefficiency of health professionals in helping patients in the cessation of smoking.

The prevalence rate found in our study was similar to studies done in Europe by Torre, et al. which found the prevalence of 29.3% and by Turhan, et al. in Turkey with a prevalence of 28.5%. But the prevalence in our study was found to be much higher than the study done by Sreeramareddy, et al. in India which found a prevalence of current smokers of only 13.1%. This finding is not satisfactory as it questions the ability of health professionals who smoke to deliver consistent messages to the patients regarding cessation and smoking-related diseases, thus it would be better if they are provided with knowledge and specific skills required for counselling.^20^

Our study found that the prevalence was higher among males compared to that of females, 23.0% vs. 7.1% respectively. This finding was similar to the study conducted by Turhan, et al. and Sreeramareddy, et al.^21,22^ The increase in prevalence in females might be due to the aggressive marketing strategy of the tobacco industry for lighter cigarettes for the woman.^22^ In contrast, a study conducted by Kusma, et al. in Berlin found no such differences.^23^

The prevalence of ever smokers in this study was found to be 49.6%, which is lower than the similar study done by Ferrante M, et al. which found ever smoker rate of much higher 74%.^20^ Similarly, a study done by Cauchi, et al. in Malta found a prevalence of 65.9%.^24^ In contrast, the study was done by Sreeramareddy, et al. found the ever smoker prevalence of only 31.7%.^25^ Several factors such as tobacco control legislation, programs, and policies, tobacco production, tobacco export and import, taxation present on tobacco products, as well as socio-economic environment play an important role in the increase in the prevalence of current smokers and ever smokers.

Our study revealed that 23.9% of the students had tried cigarettes for the first time in the later adolescence years (20-24 years) which is in contrast to a similar study done by Torre, et al. in Europe which found that 34.4% of students had tried their first cigarette in the early adolescence years (11-19 years).^26,27^ These findings could be because of poor tobacco control legislation concerning purchase by adolescents and sales.

In the study, 96.2% of the students opined that health professionals had a positive role in giving advice for the cessation of smoking. The finding is higher than that of similar studies done by Ferrante, et al. which found that only 65% of students believed that health professionals had a role in giving advice for cessation. This is a good finding, as if students give much importance to physician’s advice for the cessation of smoking, they will most likely make an effort to provide smoking prevention counseling when they themselves become the practitioners. Non-smokers thought that it was necessary for health professionals to get specific training on cessation techniques (96.2% in non-smokers vs. 79.4% in smokers), which is comparable to the findings from a study done by Ferrante M, et al. (96.6% in non-smokers vs 90.7% in smokers). This shows regardless of the smoking status of an individual, most believe it is important to get specific techniques for cessation although it is slightly lower in smokers.^20^

In our study, only 32 (28.3%) of the students actually received a formal training regarding smoking cessation this aligns with the survey done by Sreeramareddy CT, et al. which deals with country wise aggregate data, where the reporting of formal training in smoking ranged from 9.2% to 36.9%.^25^ This shows overall low rates of actual inclusion of formal training regardless of the country of the medical school, even if the students themselves believe that there is a necessity of proper training of the cessation techniques.

Passive smoke exposure remains the main issue in countries like ours. This study shows only 31.9% had no exposure while 46.9% had exposure to tobacco smoke both at home and public, exposure at only home being 5.3% and exposure at only public places being 15.9%, exposing them to more than 4 000 chemicals identified in tobacco smoke, among which at least 250 known to be harmful and more than 50 of which are known to cause cancer. Although filters and fans are used, these toxins can remain in the room for weeks as discussed in WHO report on Global Tobacco epidemic 2009.^28^

This study is a cross-sectional study that has some inevitable limitations. It gives a snapshot result of prevalence only in the specific student group selected and cannot be used to generalize among the public. However, GHPSS is a well-established tool for data collection. A self-reported questionnaire format was used although the GHPSS mainly focuses on interview-based data collection. Still, this creates a chance of social desirability bias and reporting bias. Participants were clearly informed about the anonymity and completely voluntary status of the study to minimize the biases.

## CONCLUSIONS

The prevalence of smoking among the participants is relatively high than the standard national data. Medical students are future health professionals thus should get proper cessation advice and training in proper cessation counseling techniques for future smoker patients. An inclusive curriculum and proper practical sessions are a must to aid WHO tobacco control efforts. Academic, medical, and policymaking communities should come together to create a better environment for the present medical students, to aid the prevention of non-communicable diseases.
